# Prediction of Traffic Vibration Environment of Ancient Wooden Structures Based on the Response Transfer Ratio Function

**DOI:** 10.3390/s22218414

**Published:** 2022-11-02

**Authors:** Cheng Zhang, Nan Zhang, Yunshi Zhang, Xiao Liu

**Affiliations:** 1 School of Civil Engineering, Beijing Jiaotong University, Beijing 100044, China; 2 China Electronic Engineering Design Institution Co., Ltd., Beijing 100142, China; 3 Beijing Engineering Research Center for Micro-Vibration Environmental Control, Beijing 100048, China

**Keywords:** environmental vibrations, traffic−induced vibration, vibration prediction, response transfer ratio (RTR), ancient wooden structures

## Abstract

Traffic−induced vibration is increasingly affecting people’s lives, which necessitates scrutiny of the environmental vibrations caused by traffic. This paper proposed a vibration prediction method suitable for the ancient wooden structures subjected to traffic−induced vibrations based on the multi−point response transfer ratio function. The accuracy of the proposed approach was also checked by comparing the predicted results with the measured results in the context of both the time domain and frequency domain. Subsequently, the environmental vibrations due to heavy−duty trucks passing at various speeds were measured, and the measurements were utilized as the input vibration excitation to assess the structural vibration of the Feiyun Pavilion. The structural safety was evaluated according to the “Technical specifications for protecting historic buildings against man−made vibration”. In order to meet the structural safety requirements of the Feiyun Pavilion, it is strongly recommended to limit the type and speed of vehicles in the nearby area.

## 1. Introduction

With the development of cities, environmental vibrations and noises produced by traffic are attracting more and more attention within the international scientific community [1]. The environmental vibrations caused by traffic will not only affect people’s lives and works, but also the usage of some precision instruments [2,3]. Additionally, the structural performance of buildings will deteriorate under the action of long−term traffic vibration, particularly for ancient buildings with wooden structures [4,5]. Consequently, environmental vibrations due to traffic should be further examined in the near future.

The prediction of environmental vibrations generated by traffic loading is one of the leading research directions of the understudied problem. The commonly used prediction methods for examining traffic−induced vibration mainly include theoretical analyses [6,7,8], numerical simulations [9,10,11,12], field tests [13,14,15,16,17,18], and empirical prediction formulas [19,20]. Although the theoretical analysis method leads to the exact solution in most cases, numerous assumptions and simplifications are made to the solution process; therefore, it cannot wholly reflect the actual situations of the induced vibration process. On the other hand, numerical methods have been extensively employed with the progress of computer technology; however, it is challenging to determine various parameters and complex structural modeling of a complex system problem. Commonly, the empirical prediction formula method requires a large number of measured data as the premise, and the accuracy of the prediction results will be affected by the judgment of factors.

Field tests have essentially focused on the dynamic performance analysis of several typical high−rise ancient wooden structures, while ancient wooden structures with different structural features have not been properly investigated. At the same time, most of the vibration sources are strong but short−lasting dynamic loadings such as earthquakes, while there is still a lack of research on long−lasting but micro−amplitude vibrations loadings such as traffic−induced vibration.

Therefore, this paper aimed to utilize field tests to propose a response−to−transfer ratio (RTR) vibration prediction method for ancient wooden structures under traffic loads based on the transfer function. The RTR function is the ratio of the output responses between systems. For a complex system with multiple subsystems, the overall RTR function of the system can be obtained through the RTR of each subsystem.

Similar to the transfer function vibration prediction method, the RTR vibration prediction method proposed in this paper does not need to establish the finite element model and has high calculation accuracy. At the same time, it is different from the vibration attenuation prediction method, which can only predict the magnitude of vibration energy but cannot obtain the spectral characteristics at the predicted point. The RTR vibration prediction method operates in the frequency domain. Therefore, it can accurately predict the frequency component of the vibration at the prediction point. For ancient wooden structures, the vibration of some frequencies (such as natural frequency) will cause more serious damage to the structure, so it is necessary to predict the vibration within a specific frequency band.

We took Feiyun Pavilion as the case study to verify the properties of the RTR. Then, the correctness of the RTR vibration prediction method was verified based on the field−measured data. Considering that the vibration system is a complex one with multiple subsystems, in order to reduce interference from noise vibration, the multi−point RTR was used for vibration prediction. In addition, the structural safety under the action of extreme traffic loads according to relevant codes was also evaluated.

## 2. Prediction Method of the Response Transfer Ratio Function

### 2.1. The Transfer Function and the Response Transfer Ratio Function

The transfer function is defined as the ratio of the Laplace transform of the linear system response (output) to the Laplace transform of the excitation (input) under the rest initial conditions [21]:

(1) $$T\left(s\right)=\frac{Y\left(s\right)}{X\left(s\right)}=\frac{L\left\{y\left(t\right)\right\}}{L\left\{x\left(t\right)\right\}}$$

where $T\left(s\right)$ denotes the transfer function of the linear system, $Y\left(s\right)$ and $L\left\{y\left(t\right)\right\}$ in order are the response of the system and the Laplace transform of the output, $X\left(s\right)$ and $L\left\{x\left(t\right)\right\}$ represent the excitation of the system and the Laplace transform of the input, respectively. Generally, the transfer function requires that the system can be represented by a linear time−invariant system and must be applied in the presence of the rest initial condition. It describes the differential relationships between the input and output of the system.

For complex systems with multiple subsystems, the existing interactions between the subsystems make it difficult to get the excitation of each subsystem; Therefore, it is difficult to solve the transfer function of each subsystem by exploiting the output and input. In contrast, the response of each subsystem is usually easy to get. To distinguish from the traditional transfer function, the output ratio between each pair subsystems is defined as the response transfer ratio (RTR) function:

(2) $$H_{n}=\frac{R_{n}}{R_{m}}$$

**Figure 1:**
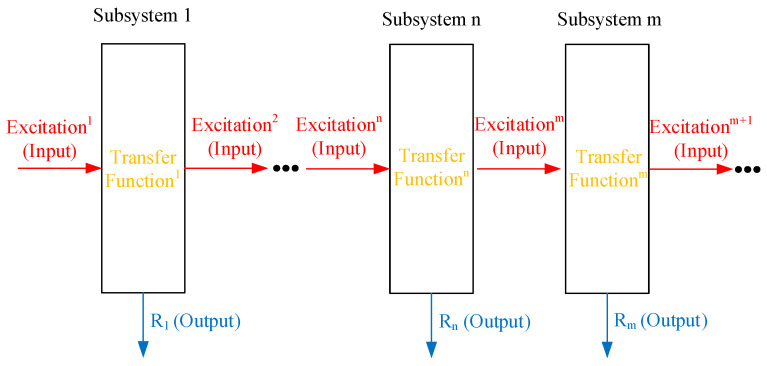
The complex systems with multiple subsystems.

In Equation (2), $H_{n}^{}$ represents the RTR function between the *n*−th subsystem and the *m*−th subsystem. As shown in Figure 1, $R_{n}$ and $R_{m}$ in order denote the output response of the *n*−th and *m*−th subsystems. Similar to the transfer function, the RTR function can describe the dynamic performance of the linear system.

For the multi−degrees−of−freedom system, the external excitation load of the system is assumed to be a simple harmonic load, that is:

(3) $$M\ddot{X}+C\dot{X}+KX=Psin\left(\omega t\right)$$

where M, C, and K in order are the mass, damping, and stiffness matrices of the system. The parameters $\ddot{X}$, $\dot{X}$, and X represent the acceleration vector, velocity vector, and displacement vector of the system, respectively, P denotes the vector of the external force applied to the system, ω is the circular frequency of the external force, and t is the time factor.

By employing the orthogonality properties of vibration modes, it is obtainable:

(4) $$M_{n}{\ddot{X}}_{n}+C_{n}{\dot{X}}_{n}+K_{n}X_{n}=P_{n}sin\left(\omega t\right)$$

A harmonic solution to Equation (4) can be sought in the following form:

(5) $$X_{n}=A_{n}sin\left(\omega_{n}t-\varphi_{n}\right)$$

where: $A_{n}=\frac{P_{n}}{K_{n}}\frac{1}{\sqrt{{\left(1-\frac{\omega }{\omega_{n}}\right)}^{2}+{\left(\frac{2\xi_{n}\omega }{\omega_{n}}\right)}^{2}}}$, $\varphi_{n}=tan^{-1}\frac{2\xi_{n}\frac{\omega }{\omega_{n}}}{1-\frac{\omega^{2}}{\omega_{n}^{2}}}$, and $\omega_{n}=\sqrt{\frac{K_{n}}{M_{n}}}$; $P_{n}$ and $\omega_{n}$ in order are the generalized load and natural frequency associated with the *n*−th vibration mode; $M_{n}$ and $K_{n}$ represent the generalized mass and stiffness corresponding to the *n*−th mode, respectively, and $\xi_{n}$ denotes the generalized damping ratio. By employing the superposition of modes in view of Equation (5), we can arrive at:

(6) $$X=\overset{N}{\underset{n=1}{\sum }}\phi_{n}^{T}X_{n}=\overset{N}{\underset{n=1}{\sum }}\left[\phi_{n}^{T}A_{n}sin\left(\omega t-\varphi_{n}\right)\right]$$

where $\phi_{n}^{}$ denotes the vector pertinent to the *n*−th vibration mode, $X_{n}$ represents the generalized mode participation coefficient, while $A_{n}$ and $\varphi_{n}$ are their corresponding constants. 

According to Equation (6), in the multi−degrees−of−freedom system under the action of single frequency vibration excitation, the steady−state response of each degree−of−freedom is still evaluated based on a single frequency, and the vibration frequency is the same as the excitation one.

When the input load in Equation (4) varies in constant λ times, the resulting solution is readily resulted by:

(7) $$X_{n}=\lambda A_{n}sin\left(\omega t-\varphi_{n}\right)$$

(8) $$X=\overset{N}{\underset{n=1}{\sum }}\phi_{n}^{T}X_{n}=\overset{N}{\underset{n=1}{\sum }}\left[\phi_{n}^{T}\lambda A_{n}sin\left(\omega t-\varphi_{n}\right)\right]$$

According to Equation (8), when the ratio of the input load remains unchanged in various frequency bands, the ratio of the output acceleration also remains unchanged in the corresponding frequency band.

**Figure 2:**
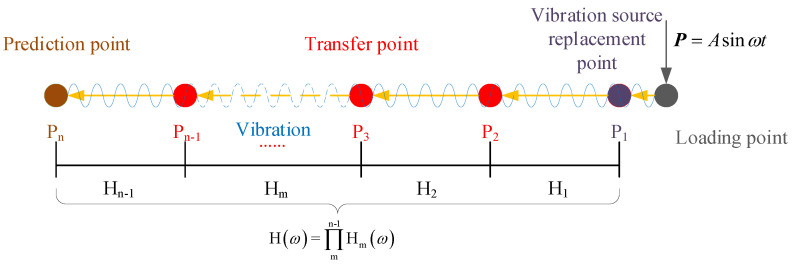
Schematic representation of the main procedure of the multipoint RTR.

Therefore, for the multi−point RTR, input a simple harmonic force P=Asinωt at the loading point, the RTR function between the points $P_{n-1}$ and $P_{n}$ is represented by $H_{n-1}$. As a result, the RTR function between the vibration source replacement point $P_{1}$ and the vibration prediction point $P_{n}$ can be expressed by:

(9) $$H\left(\omega \right)=\overset{n-1}{\underset{m}{\prod }}H_{m}\left(\omega \right)$$

where $H_{m}$ denotes the RTR between point $P_{m+1}$ and point $P_{m}$, *n* represents the number of transfer points on the transfer path. The calculation diagram of the multipoint RTR has been demonstrated in Figure 2.

### 2.2. Vibration Prediction Based on the RTR

For the environmental vibration caused by traffic, when a vehicle passes the road, the roadside vibration acceleration, $x\left(t\right)$, and the acceleration at the prediction point, $y\left(t\right)$, can be recorded simultaneously. Considering the calculation efficiency and accuracy, we then proceed in dividing the accelerations $x\left(t\right)$ and $y\left(t\right)$ according to the frequency bandwidth of the one−third octave. The result $X\left(t,f\right)$ and $Y\left(t,f\right)$ of such a division is the corresponding acceleration time−history data in each frequency band, the so−called octave time−history data in the present study. Specific processes for octave time−history data are as follows: 1. Take the Fourier transform of the acceleration data from the time domain to the frequency domain; 2. According to the one−third octave band, band−pass filtering is performed in turn to select the acceleration data in each frequency band; 3. Finally, the inverse Fourier transform is performed on the selected acceleration data in each frequency band to obtain the acceleration time−history data in the corresponding frequency band, which is called octave time−history data.

Subsequently, the ratio of maximum value of the octave time−history data, obtainable from the corresponding frequency band $MAX\left(X\left(t,f\right)\right)$ and $MAX\left(Y\left(t,f\right)\right)$, are defined as the amplitude−RTR. Mathematically, it is stated by:

(10) $$H{\left(f\right)}_{\mathrm{MAX}}=\frac{MAX\left(X\left(t,f\right)\right)}{MAX\left(Y\left(t,f\right)\right)}$$

Further, the ratio of the acceleration root−mean−square (RMS) of the octave time−history data $RMS\left(X\left(t,f\right)\right)$ and $RMS\left(Y\left(t,f\right)\right)$ in the corresponding frequency band is defined as the RMS−RTR, which is calculated by:

(11) $$H{\left(f\right)}_{\mathrm{RMS}}=\frac{RMS\left(X\left(t,f\right)\right)}{RMS\left(Y\left(t,f\right)\right)}$$

Using hammer excitation at the same excitation point, the roadside vibration acceleration time−history $x^{\prime }\left(t\right)$ and the acceleration time−history $y^{\prime }\left(t\right)$ at the prediction point can be simultaneously recorded. According to the one−third octave calculation method, the octave time−history data $X^{\prime }\left(t,f\right)$ and $Y^{\prime }\left(t,f\right)$ are computed, and similar to Equations (10) and (11), the RTR function of the roadside−prediction point acted upon by the hammering excitation is evaluated according to Equations (12) and (13):

(12) $$H\prime {\left(f\right)}_{\mathrm{MAX}}=\frac{MAX\left(X^{\prime }\left(t,f\right)\right)}{MAX\left(Y^{\prime }\left(t,f\right)\right)}$$

(13) $$H\prime {\left(f\right)}_{\mathrm{RMS}}=\frac{RMS\left(X^{\prime }\left(t,f\right)\right)}{RMS\left(Y^{\prime }\left(t,f\right)\right)}$$

It is assumed that the RTR function calculated by the hammer excitation test can be exploited to replace the transfer ratio function of the roadside−prediction point under the action of the traffic excitation. Thereby,

(14) $$H{\left(f\right)}_{\mathrm{MAX}}\approx H\prime {\left(f\right)}_{\mathrm{MAX}}$$

(15) $$H{\left(f\right)}_{\mathrm{RMS}}\approx H\prime {\left(f\right)}_{\mathrm{RMS}}$$

The roadside−prediction point RTR functions $H^{\prime }{\left(f\right)}_{RMS}$ and $H^{\prime }{\left(f\right)}_{MAX}$ can be measured by the hammer excitation and then combined with the traffic−induced roadside acceleration octave time−history data $X_{pre}\left(t,f\right)$. Subsequently, the output octave time−history data at the prediction point can be calculated through the following relations:

(16) $$Y_{pre}\left(t,f\right)=X_{pre}\left(t,f\right)/H{\left(f\right)}_{MAX}\approx X_{pre}\left(t,f\right)/H\prime {\left(f\right)}_{MAX}$$

(17) $$Y_{pre}\left(t,f\right)=X_{pre}\left(t,f\right)/H{\left(f\right)}_{RMS}\approx X_{pre}\left(t,f\right)/H\prime {\left(f\right)}_{RMS}$$

Finally, by superimposing the octave time−history data $Y_{pre}\left(t,f\right)$ associated with each frequency band, the acceleration time−history data $y_{pre}\left(t\right)$ at the predicted point can be evaluated as follows:

(18) $$y_{pre}\left(t\right)=\underset{f}{\sum }Y_{pre}\left(t,f\right)$$

**Figure 3:**
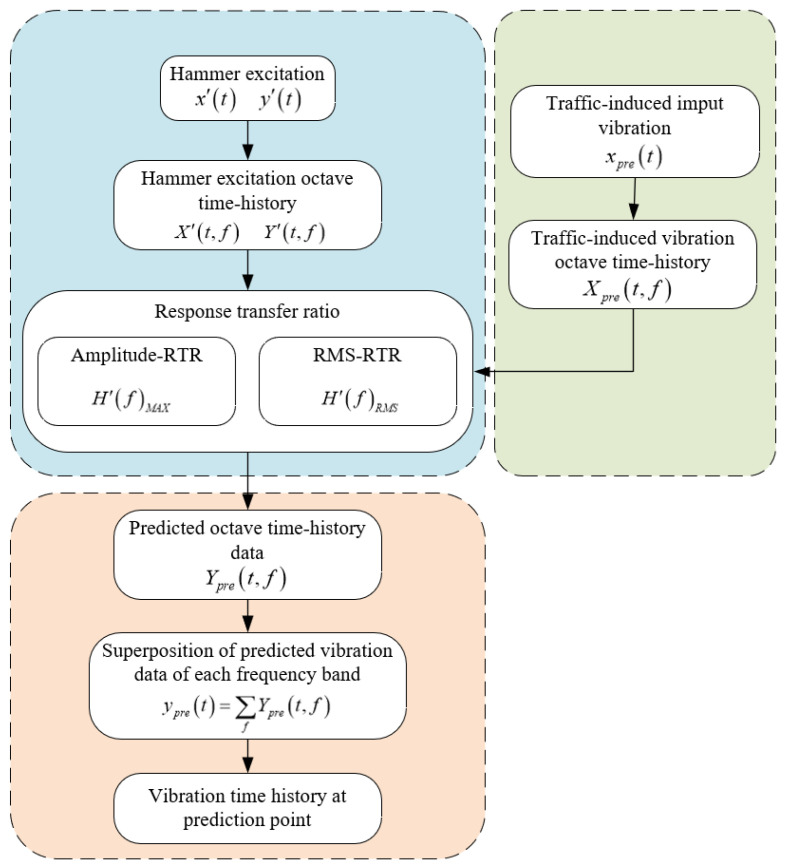
The flowchart of traffic environmental vibration prediction based on the measured RTR.

The proposed traffic−induced vibration prediction method has been flowcharted in Figure 3.

The vibration prediction method based on the RTR is as follows: Firstly, the response transfer ratio between the roadside and the prediction point is obtained through the artificial excitation vibration test; Then, collect the roadside environmental vibration caused by traffic load; Finally, the measured traffic−induced vibration is used as the excitation to calculate the vibration response of the prediction point by calculated RTR.

### 2.3. Field Test of the RTR Function

Theoretically, the transfer function is only related to the tested object, representing its inherent attribute, and it does not change with different external incentives. In order to verify whether the proposed RTR function also satisfies this characteristic, we designed two groups of hammer excitation experiments with a hammer weight of 30 kg. One group was oriented to control the hammer to fall from different heights, and only the change of the excitation energy was allowed without altering the excitation frequency. The other group dropped the hammer at the same height, and placed rubber, wood, and steel blocks at the landing point of the hammer. Therefore, the latter group was aimed at changing the input load spectrum characteristics, without altering the excitation energy. The test conditions are also presented in Table 1.

**Figure 4:**
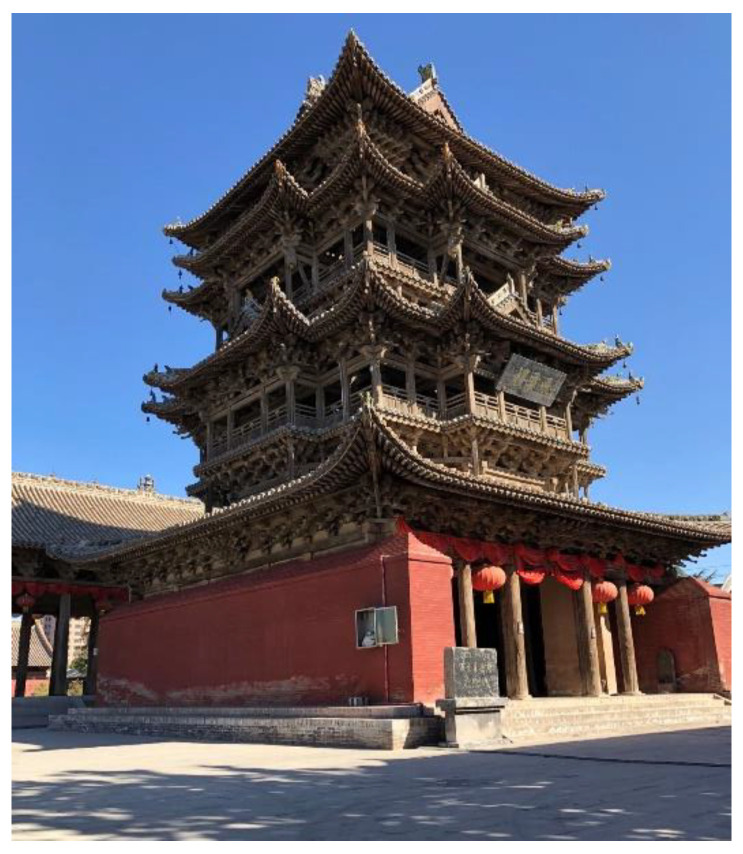
The Feiyun Pavilion.

As part of research series, this paper takes the Feiyun Pavilion as the case study. The Feiyun Pavilion (see Figure 4) is a purely wooden building in the Yuan (1271–1368 AD) and Ming Dynasty (1368–1683 AD) styles. It is located within the Dongyue temple in Wanrong County, Yuncheng City, Shanxi Province, China. The entire building is mainly made of wood, and the structural connection uses mortise and tenon joints without any metal components. The pavilion has three floors on the outside and five floors inside. The total height of the building is about 23 m.

**Figure 5:**
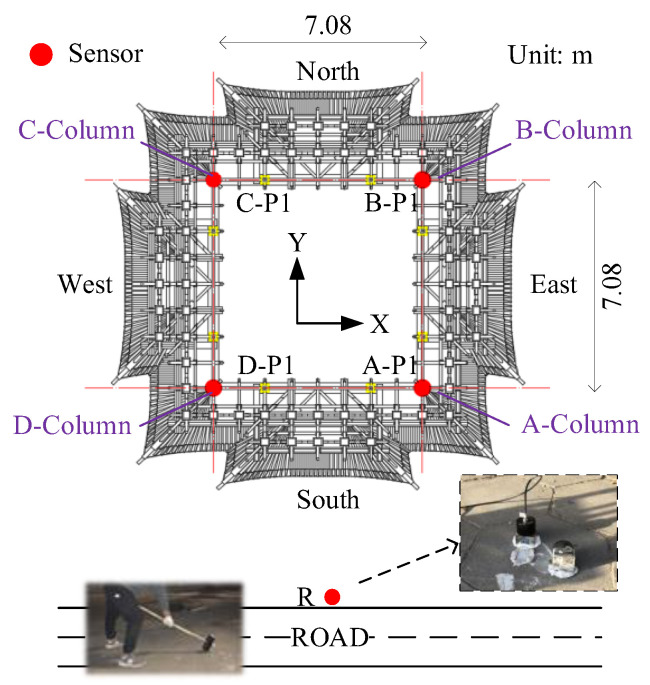
The horizontal layout of the measurement points. (Field Test of the RTR Function).

**Figure 6:**
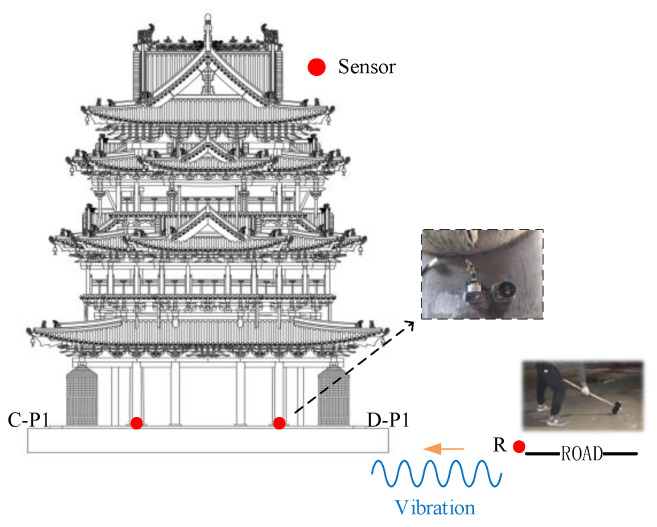
The vertical layout of the measurement points. (Field Test of the RTR Function).

The layout of the measurement points is demonstrated in Figure 5 and Figure 6. The excitation point of the drop weight is on the road, which is ten meters away from the south side of the Feiyun Pavilion. The vibration source replacement point (R) is arranged at 1m perpendicular to the road, which is to collect the output response of road traffic subsystem. On the first floor of the Feiyun Pavilion near the four through columns, first floor measuring points (A−P1, B−P1, C−P1, D−P1) are set up and employed to measure the output acceleration of the system under the action of hammering excitation. In order to measure the RTR in three directions, all measuring points should be equipped with acceleration sensors in horizontal east−west, horizontal north−south direction, and vertical direction. The sampling frequency is set equal to 512 Hz.

The equipment utilized in the test includes an INV3020C synchronous data acquisition system with 28 channels, and 15 uniaxial (10 horizontal and 5 vertical) 941b acceleration sensors. Before the test, the acceleration sensors are appropriately calibrated for consistency and sensitivity.

#### 2.3.1. Variation of the RTR Function with Excitation Vibration Energy

The artificial excitation vibration was applied to the system by using a free−falling weight. By controlling the hammer falling from heights of 50–70 cm with an increment of 5 cm, we examined whether the RTR of the system would change due to the same spectral characteristics but with various vibrational energies. Considering that the environmental vibration caused by traffic is mainly low−frequency vibration, we mainly analyzed the vibration below 80 Hz.

**Figure 7:**
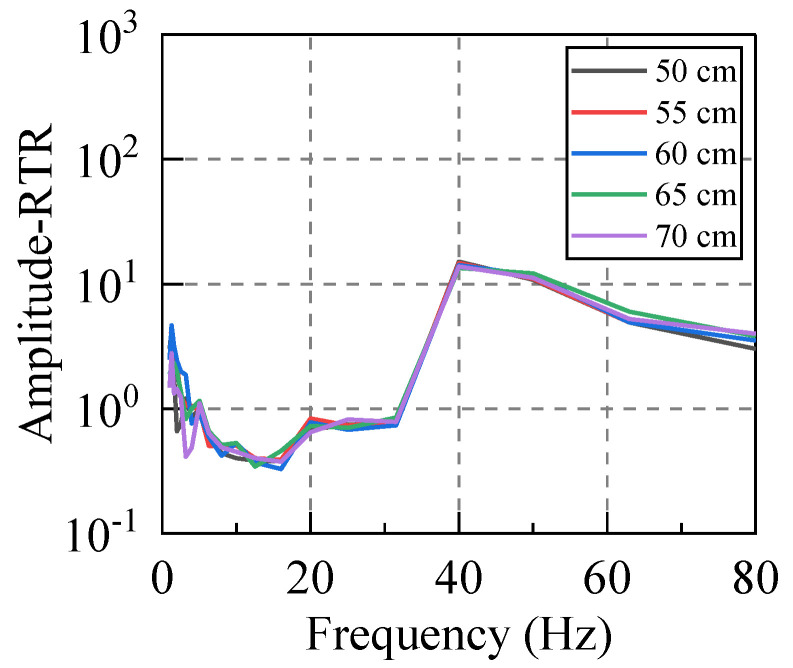
Amplitude−RTR. (Different excitation heights).

**Figure 8:**
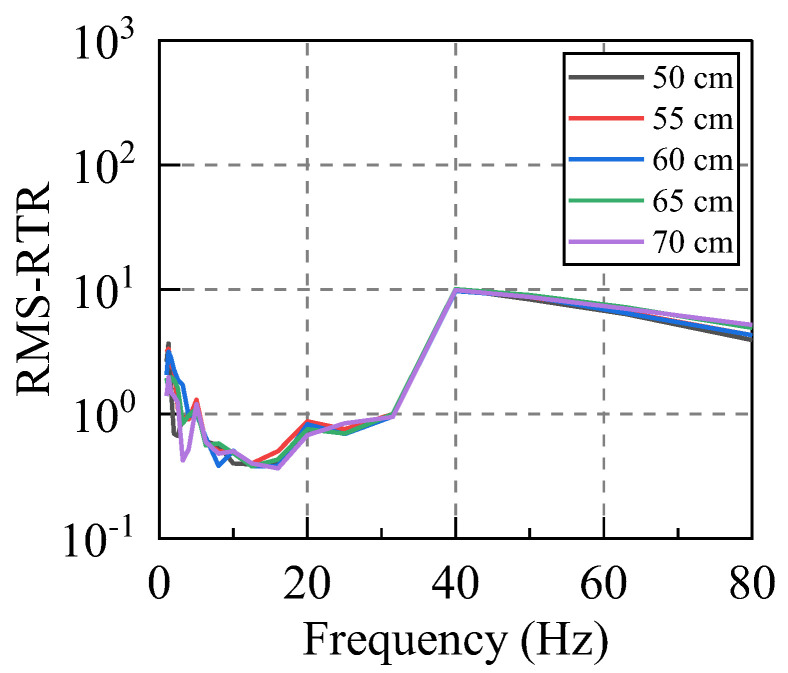
RMS−RTR. (Different excitation heights).

According to Equations (12) and (13), the RTRs of the system subjected to different levels of the input energy were calculated. For this purpose, at least five sets of valid hammer vibration data were collected for each working condition, and the RTR from the vibration source replacement point (R) to the first floor measuring point (A−P1, B−P1, C−P1, D−P1) was calculated due to each hammering excitation. Subsequently, the average value of five groups of RTRs in the same direction under the same working condition was taken as the RTR in this direction under this working condition. Taking the horizontal east−west direction (X direction) as an example, the amplitude−RTR and the RMS−RTR under different hammering excitations were evaluated, and the obtained results are graphed in Figure 7 and Figure 8.

Firstly, the RTR functions calculated by the two distinct methods were compared. It can be seen that although their calculation bases are completely different, the discreteness of the two approaches is small for frequencies below 80 Hz, and the variation laws in terms of the frequency are the same. The RTR functions, which are calculated by the same method, are also compared. The RTR varies slightly when the input vibration energy is significantly different, and the consistency is satisfactory in the frequency range of 5–63 Hz. Therefore, both methods can appropriately calculate the RTR function of the system, and its value is independent of the input vibration energy.

#### 2.3.2. Variation of the RTR Function with Excitation Vibration Frequency

**Figure 9:**
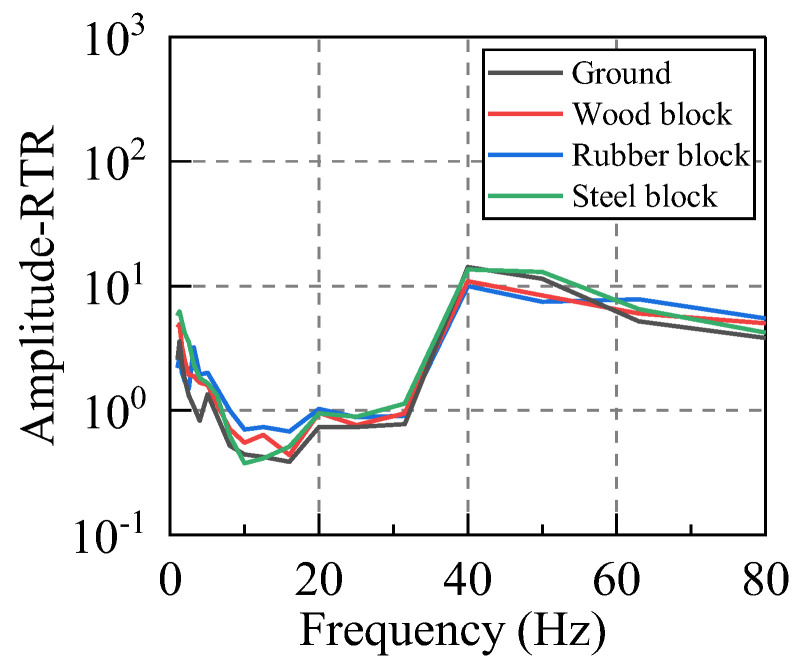
Amplitude−RTR. (Different cushion blocks).

**Figure 10:**
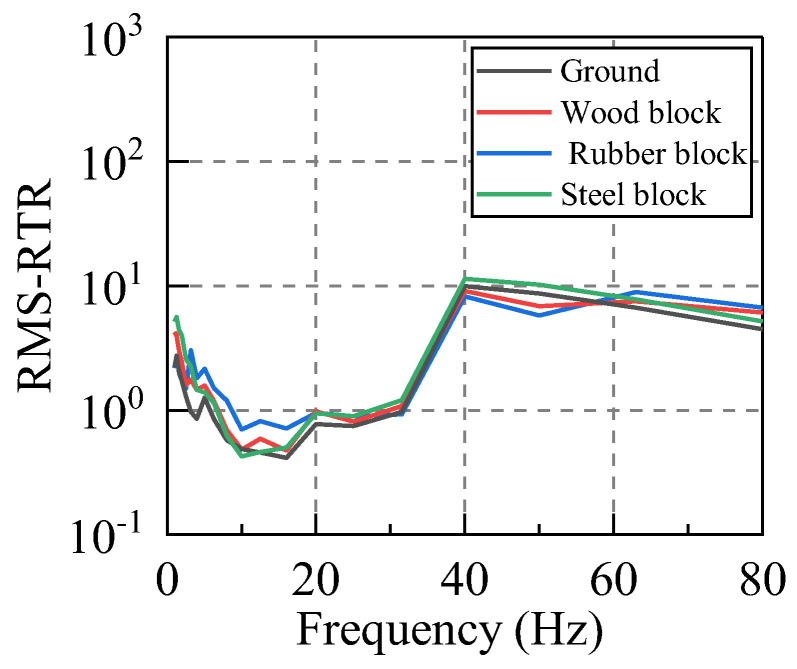
RMS−RTR. (Different cushion blocks).

In order to input the same vibration energy to the system, we let the hammer have a free fall from 60 cm. In addition, steel plate, wood plate, and rubber plate are placed at the hammering point in order to apply vibrations with the same excitation energy but different spectral characteristics to the system. The RTR function between vibration source replacement point to the first floor measuring point was calculated according to Equations (12) and (13). Taking the X direction as an example, the calculation results for this case are illustrated in Figure 9 and Figure 10.

By comparing the RTR acted upon by the excitation with different spectrum characteristics, it can be seen that the calculation results of the RTR in the presence of various working conditions are relatively consistent. Therefore, the different input excitation spectrum has little influence on the RTR of the same system, and this characteristic is more prominent for frequencies in the range of 5–63 Hz. The RTR is independent of the input vibration spectral characteristics.

Through the calculation and analysis of the RTR under various working conditions, it can be inferred that the RTR is only affected by the dynamic characteristics of the structural system. It is the inherent attribute of the structural system, and it will not change for different input excitations. It implies that the application of the hammering excitation to calculate the RTR of the system can be utilized as an appropriate replacement of the RTR under the action of the traffic excitation. This indicates that the Equations (14) and (15) are reasonable.

## 3. Vibration Prediction Based on the Measured RTR Function

### 3.1. Introduction to the On−Site Dynamic Test

**Figure 11:**
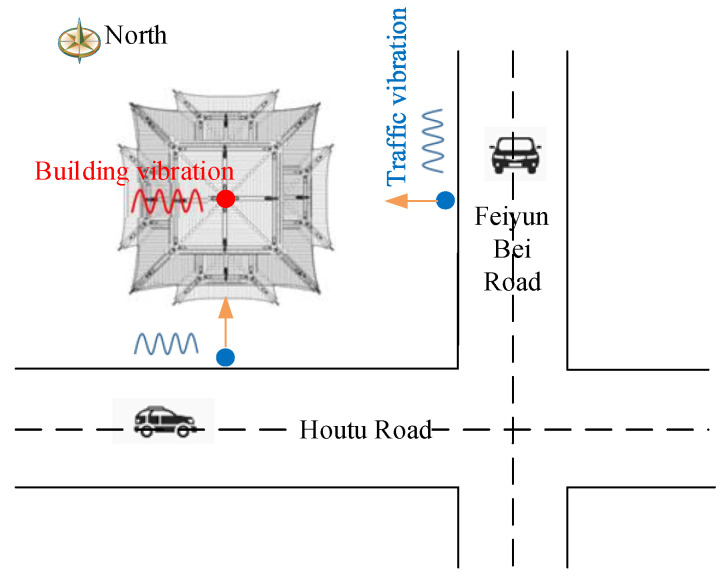
Schematic representation of the traffic environment around the Feiyun Pavilion.

As demonstrated in Figure 11, Feiyun Pavilion is only 10 m away from Houtu road in the south and close to Feiyun Bei road in the east. The long−term wind, rain erosion, and the impact of traffic vibration have made damages to Feiyun Pavilion up to a certain extent. Therefore, it is necessary to predict the vibration of the Feiyun Pavilion under the action of traffic loading.

**Figure 12:**
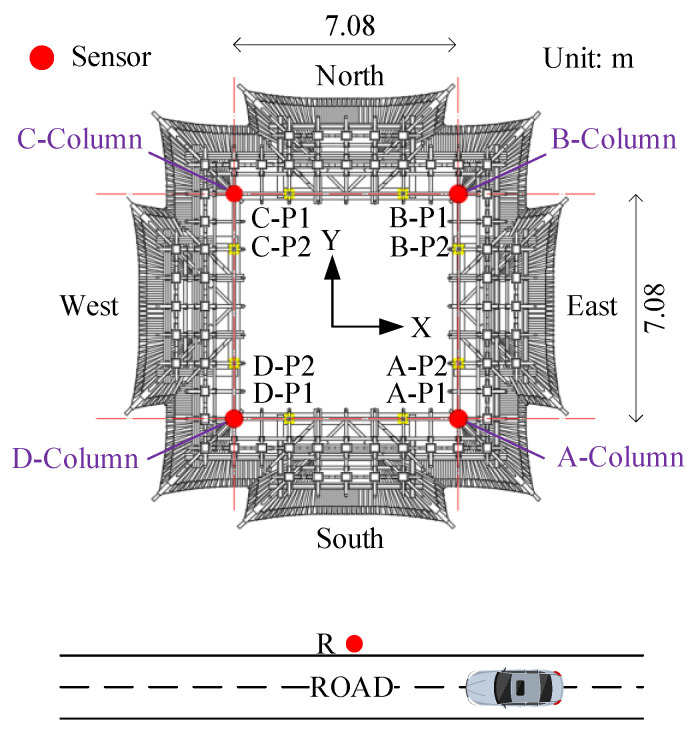
The layout plan of the measurement points.

**Figure 13:**
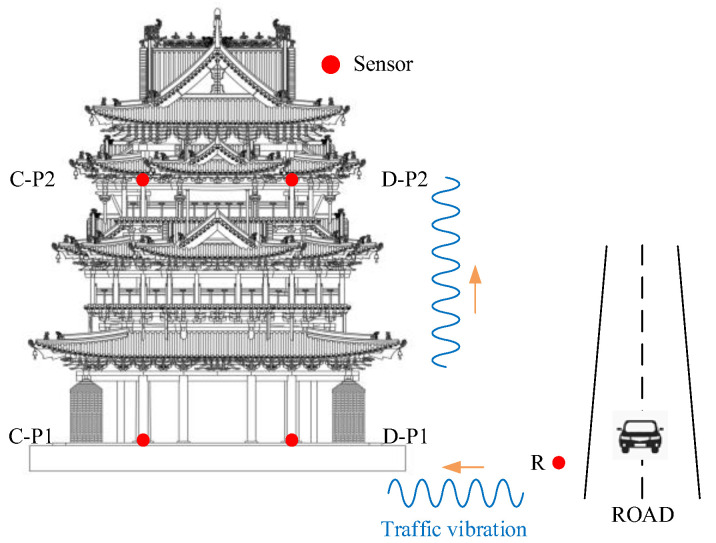
The vertical layout of the measurement points.

Considering the whole media of the vibration transmission is composed of the road, soil, foundation, and superstructure. The measuring points of such a media are particularly arranged as presented in Figure 12 and Figure 13. This is somehow similar to the measurement point arrangement in the “Field test of the RTR function”. In addition to arranging the vibration source replacement point (R) and the first floor measuring points (A−P1, B−P1, C−P1, and D−P1), it is also necessary to arrange the third floor measuring points (A−P2, B−P2, C−P2, and D−P2) at the top of the four corner columns. The third floor measuring points are also exploited to collect the vibration at the highest point of the structure. At the same time, the vibration data of the third floor measuring point can also evaluate the structural safety of the Feiyun Pavilion according to the relevant specifications.

Due to the increase of the measuring points, we choose the following equipment to collect vibration data: INV3020C synchronous data acquisition system with 28 channels and 27 uniaxial (18 horizontal and 9 vertical) 941b acceleration sensors.

### 3.2. The On−Site Test of the Multi−Point RTR

Let us take the measuring point in column D as an example. Firstly, the free−fall hammer is utilized to input excitation vibration to the system from a height of 60 cm, on the Houtu road. Then, the multi−point RTR function between the vibration source replacement point (R), the first floor measuring point (D−P1), and the third floor measuring point (D−P2) were calculated according to the acceleration data. After that, under the action of the traffic−induced vibration, the measured environmental vibration at point R was employed as the excitation, and the multi−point RTR calculated by the hammering test was employed to predict the traffic−induced vibration at the third floor measuring point (D−P2). Finally, the effectiveness of the proposed vibration prediction method was verified by comparing the measured vibration of the third floor measuring point (D−P2) with the predicted vibration.

**Figure 14:**
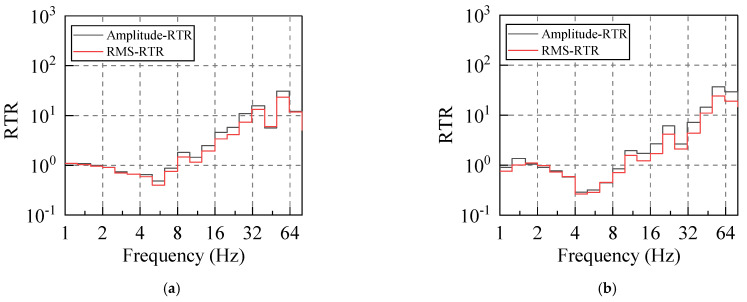

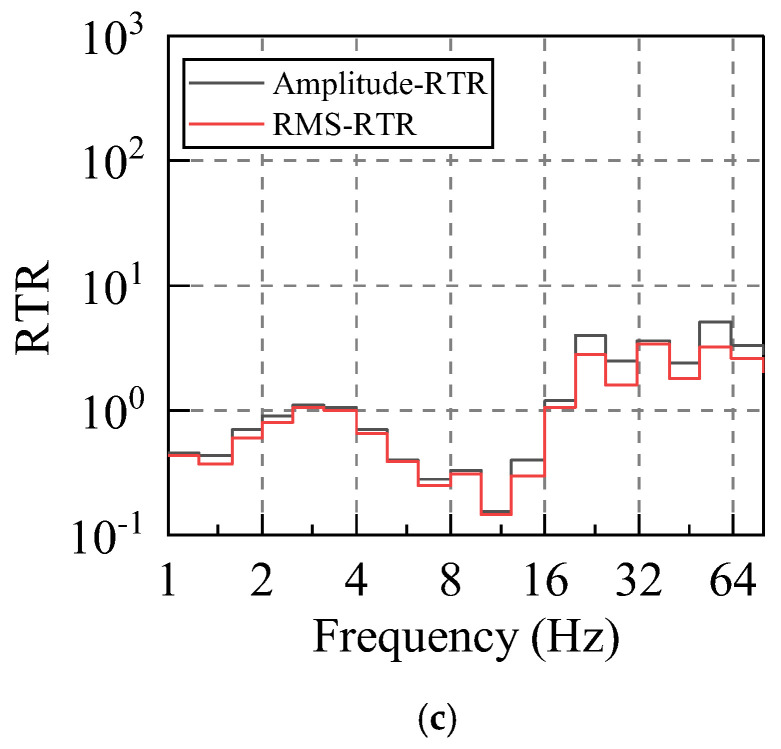
The measured RTR: (**a**) vertical direction; (**b**) east−west direction; and (**c**) south−west direction.

According to Equation (9), the collected acceleration data were calculated to obtain the multi−point (R to D−P2) RTR. The plotted results are demonstrated in Figure 14.

### 3.3. Structural Vibration Prediction due to Traffic−Induced Vibration

**Figure 15:**
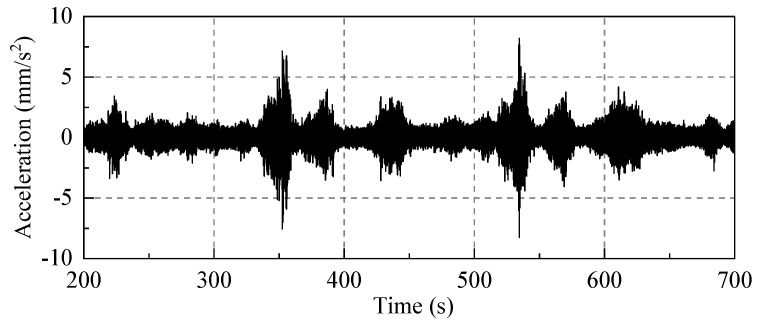
The time−history of acceleration of the third floor measuring point (D−P2) in the east−west direction.

In order to ensure that a sufficient amount of traffic vibration data were collected, the sampling duration and frequency in order were set to 1200 s and 512 Hz. Due to the traffic−induced vibration, the partial acceleration data of the third floor measuring point (D−P2) in the horizontal east−west direction (X direction) has been presented in Figure 15.

**Figure 16:**
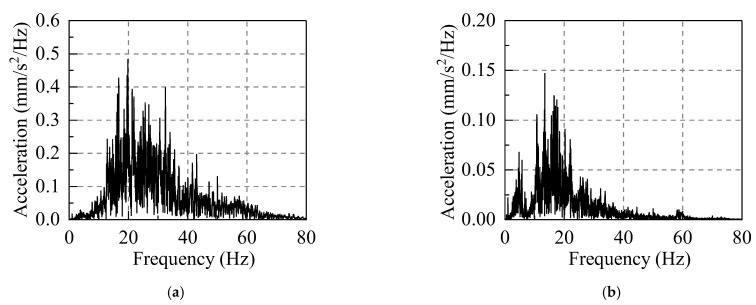
Acceleration spectrums: (**a**) spectrum of the vibration source replacement point (R); and (**b**) spectrum of the third floor measuring point (D−P2).

A representative traffic−induced vibration data segment (520s−570s) was selected for fast Fourier transform (FFT) analysis. The vibration data in the frequency−domain were then analyzed, as shown in Figure 16.

The vibration at the vibration source replacement point (R) is mainly low−frequency vibration below 80 Hz, of which 5 Hz−60 Hz represents its excellent frequency band. When the vibration is transmitted from the vibration source replacement point (R) to the third floor measuring point (D−P2), its spectrum characteristics have considerably altered: the vibration with a frequency above 40 Hz is greatly attenuated, and the vibration is mainly low−frequency vibration with frequencies in the range of 5−40 Hz. Therefore, this frequency interval is defined as the prediction and analysis frequency band. In the vibration prediction, the acceleration for the frequency interval 5–40 Hz will be mainly calculated.

**Figure 17:**
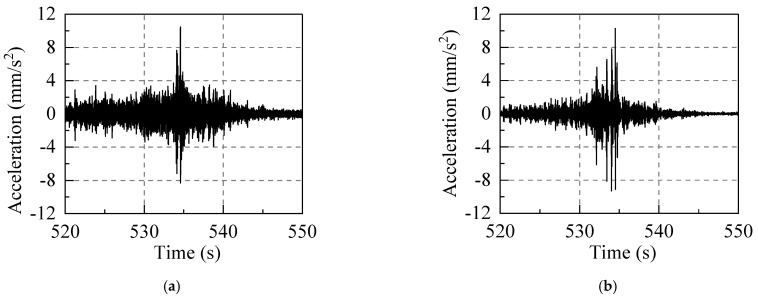

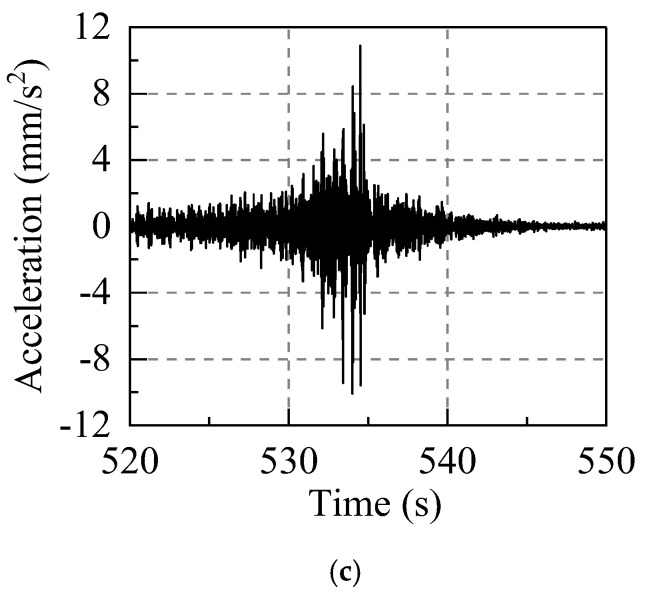
Acceleration time−history of the third floor measuring point (D−P2): (**a**) traffic−induced acceleration in third floor measuring point (D−P2); (**b**) third floor measuring point (D−P2) acceleration time−history prediction based on the amplitude−RTR, and (**c**) third floor measuring point (D−P2) acceleration time−history prediction based on the RMS−RTR.

According to the RTR, the traffic−induced vibration data at the third floor measuring point (D−P2) can be predicted. The time−history data of the prediction results are presented in Figure 17:

### 3.4. Evaluate Prediction Accuracy

Because the phase difference between the measuring points is ignored in the prediction process, the prediction accuracy is generally evaluated by statistical indicators.

The square of the root−mean−square (RMS) can be employed to quantify the average vibration energy at each measurement point [22]. Hence, this factor is capable of evaluating the vibration response produced by traffic vibrations. The RMS value of a discrete−time signal is defined by:

(19) $$a_{RMS}=\sqrt{\frac{\overset{N}{\underset{i=1}{\sum }}a^{2}\left(i\right)}{N}}$$

where *a* denotes the measured acceleration, and *N* represents the number of data points analyzed. 

The computed results of the maximum and RMS at the third floor measuring point (D−P2) during the analysis period have been presented in Table 2.

By comparing the measured data and the predicted one, it can be seen that the amplitude−RTR can be utilized to predict the vibration of the third floor measuring point (D−P2) subjected to traffic−induced vibration. The difference between the maximum acceleration and the real value is reported as 1.8%, and the relative discrepancy between the predicted acceleration RMS and the real value is about 6.1%. Employing the RMS−RTR to predict the vibration of the third floor measuring point, the generated relative error between the maximum acceleration and the real value is 4.5%, and the resulting error between the RMS and the real value is about 3.1%. The main reasons for the produced error are as follows: (1) The signal−to−noise ratio of the acceleration data due to the traffic−induced vibration is poor; (2) The environment of the on−site test is complex, so the measured signal can be easily disturbed; (3) Only the excellent frequency band is predicted, while the vibration energy in other frequency bands is overlooked. 

**Figure 18:**
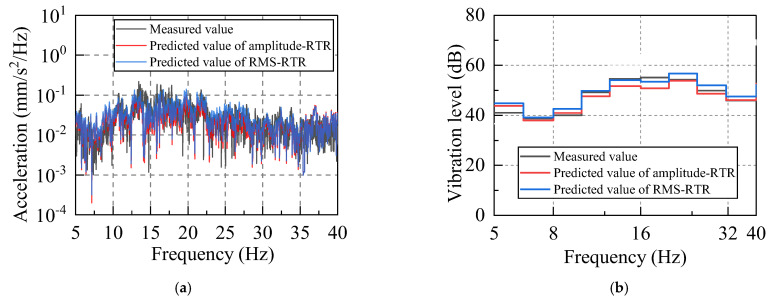
The frequency−domain calculation results of the measured and predicted values: (**a**) Power spectra density, (**b**) 1/3−octave.

The accuracy of the prediction results was also evaluated in the frequency−domain. The spectrum, 1/3−octave of the measured data, and predicted data of the third floor measuring point are calculated. The predicted results have been now illustrated in Figure 18. The frequency−domain analysis revealed that both the predicted and measured results can have good consistency in the predicted analysis frequency band. 

In both the time−domain and frequency−domain, the application of the measured RTR function for examining the vibration of ancient wooden structures exhibited a high prediction accuracy due to the traffic−induced vibration.

## 4. Prediction and Evaluation of Structural Safety of the Feiyun Pavilion due to Extreme Traffic Loading

**Figure 19:**
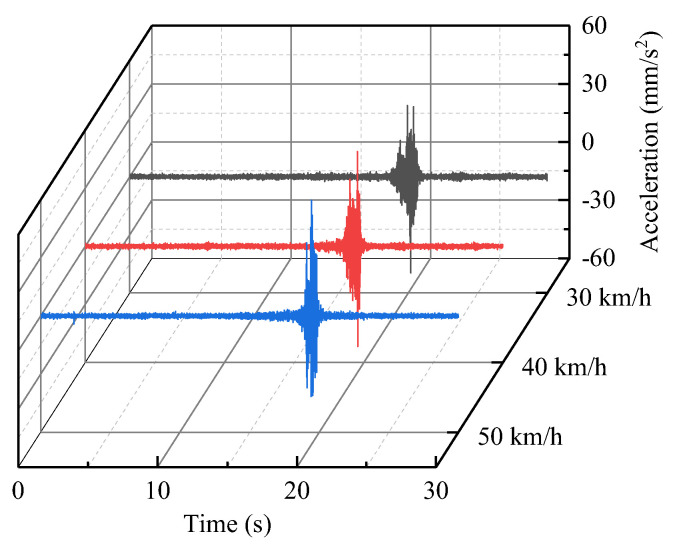
The acceleration of the vibration source replacement point (horizontal east−west direction).

In order to explore the vibration of the Feiyun Pavilion under the action of extreme traffic loading and evaluate its structural safety according to the prediction results, we performed the excitation test of heavy−duty trucks (the vehicle’s weight is 40 tons) on a road similar to the Houtu road. The acceleration data (horizontal north−south, horizontal east−west, and vertical components) were collected at the vibration source replacement point when the vehicle speeds are 30, 40, and 50 km/h. The sampling frequency is 512 Hz and the recording time period is 30 s. The horizontal east−west acceleration data at the vibration source replacement point have been plotted in Figure 19.

According to the “Technical specifications for protection of historic buildings against man−made vibration” [23], the allowable vibration of ancient structures should be controlled by the horizontal vibration velocity at the highest point of the structure. Therefore, the limit of horizontal vibration velocity at the highest point of the Feiyun Pavilion is 0.18 mm/s.

**Figure 20:**
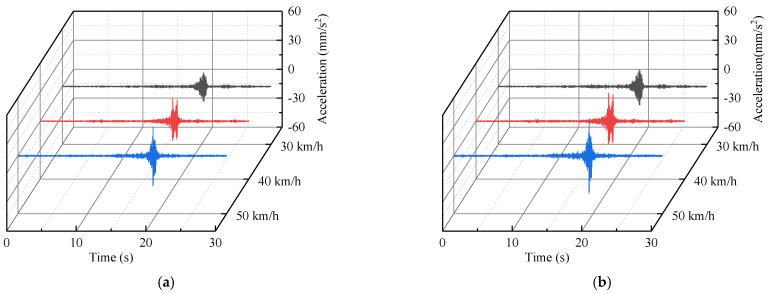
Predicted acceleration time−history of the third floor measuring point (D−P2) under the heavy−duty truck (horizontal east−west direction): (**a**) third floor measuring point (D−P2) acceleration time−history prediction based on the amplitude−RTR; and (**b**) third floor measuring point (D−P2) acceleration time−history prediction based on the RMS−RTR.

**Figure 21:**
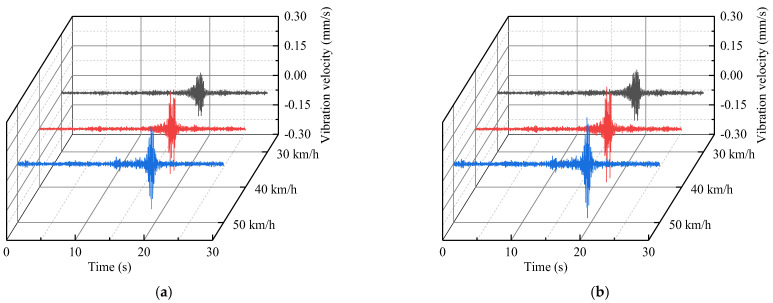
Predicted velocity time−history of the third floor measuring point (D−P2) under the heavy−duty truck (horizontal east−west direction): (**a**) third floor measuring point (D−P2) vibration velocity time−history prediction based on the amplitude−RTR; and (**b**) third floor measuring point (D−P2) vibration velocity time−history prediction based on the RMS−RTR.

According to the proposed vibration prediction method, the vibration acceleration of third floor measuring point (D−P2) caused by a heavy−duty truck can be obtained, based on the amplitude−RTR and RMS−RTR (Figure 20). The predicted vibration velocity of third floor measuring point (Figure 21) was obtained by integrating the predicted acceleration data in the frequency−domain. The maximum predicted vibration velocity of the third floor measuring point have been provided in Table 3.

The calculation results revealed in Table 3 show that even when the heavy−duty truck passes through the Feiyun pavilion at a minimum test speed of 30 km/h, the structural vibration has exceeded the specification limit, which will pose a threat to its structural safety. Therefore, it is suggested to limit the type and speed of vehicles in the Feiyun Pavilion areas for protecting the ancient wooden structures.

## 5. Conclusions

(1) The multipoint RTR was derived, and showed that the RTR function is the inherent property of the structure and does not alter with excitation load energy and frequency;
(2) Based on the RTR function, a vibration prediction method suitable for the ancient wooden structures subjected to traffic−induced vibration was proposed. By comparing with the measured data, the prediction results represented a good accuracy in both the time and frequency domains;
(3) The structural vibration of the Feiyun Pavilion due to extreme traffic loads was predicted, and the corresponding structural safety was evaluated according to the “Technical specifications for protection of historic buildings against man−made vibration”. The calculation results reveal that in the Feiyun Pavilion area, it is necessary to restrict the type and speed of vehicles to protect the ancient wooden structures from traffic−induced vibrations.

## Figures and Tables

**Table 1:** Working conditions of the drop weight test: (**a**) Different excitation heights, (**b**) Different cushion blocks.

(a)							
Test Name	Working Condition					Measuring Point Location	
	Condition 1	Condition 2	Condition 3	Condition 4	Condition 5	Vibration Source Replacement Point R	First Floor Measuring Point
Drop weight height (cm)	50	55	60	65	70	R	A−P1 B−P1 C−P1 D−P1
**(b)**							
**Test Name**	**Working Condition**					**Measuring Point Location**	
	**Condition 1**	**Condition 2**		**Condition 3**	**Condition 4**	**Vibration Source Replacement Point R**	**First Floor Measuring Point**
Cushion block	−	Wood block		Rubber block	Steel block	R	A−P1 B−P1 C−P1 D−P1

**Table 2:** Comparison between the predicted and measured acceleration data at the third floor measuring point (D−P2).

	Maximum Acceleration in Time−Domain (mm/s^2^)	RMS of Acceleration in Time−Domain (mm/s^2^)
Measured value	10.47	0.97
Predicted value of amplitude−RTR	10.28	0.91
Predicted value of RMS−RTR	10.89	0.94

**Table 3:** The maximum predicted vibration velocity of the third floor measuring point (D−P2) under the action of heavy−duty trucks.

Vehicle Speed (km/h)	The Maximum of Vibration Velocity Based on Amplitude−RTR		The Maximum of Vibration Velocity Based on RMS−RTR	
	East−West Direction (mm/s)	North−South Direction (mm/s)	East−West Direction (mm/s)	North−South Direction (mm/s)
30	0.12	0.30	0.14	0.31
40	0.23	0.69	0.27	0.75
50	0.24	2.03	0.28	2.13

## Data Availability

The data presented in this study are available upon request from the corresponding author.
